# Mycobacterium abscessus Pneumonia in Severe Alcoholism

**DOI:** 10.7759/cureus.26251

**Published:** 2022-06-23

**Authors:** Sudeep Acharya, Shamsuddin Anwar, Yelizaveta Medina, Sakura Thapa, Allison Glaser

**Affiliations:** 1 Internal Medicine, Northwell Health, New York, USA; 2 Infectious Diseases, Tufts Medical Center, Boston, USA; 3 Internal Medicine, Greencity Hospital, Kathmandu, NPL; 4 Internal Medicine, Staten Island University Hospital, New York, USA

**Keywords:** immunocompromised individuals, non-tuberculosis pneumonia, pulmonary cavitary lesion, non tuberculous mycobacteria, mycobacterium abscessus

## Abstract

Non-tuberculous mycobacterial infections (mycobacterium other than *Mycobacterium tuberculosis* and *Mycobacterium leprae*) are organisms that are commonly found in the environment such as water, soil, and dust. They may form difficult to eliminate biofilms and have been reported to cause significant infections in humans, especially in immunocompromised hosts. This article describes an interesting case of *Mycobacterium abscessus* in the lung.

## Introduction

In the United States, *Mycobacterial tuberculous* infection is not commonly seen, and the rate of infection has substantially decreased (incidence of 2.4 cases per 100,000 persons in 2021 from 12.2 in 1980 according to the CDC website). The incidence of nontuberculous mycobacterial (NTM) infection on the other hand is rapidly increasing. A relatively recent study suggested an increase in incidence from 2.5 cases per 100,000 persons in the 1980s to 15.2 cases per 100,000 persons by 2013 in the United States [1]. A nontuberculous mycobacterial pulmonary disease is typically described in patients with underlying parenchymal lung disease such as cystic fibrosis, bronchiectasis, or prior history of tuberculosis as well as immunocompromised patients, such as those with advanced HIV or other forms of long-term immunosuppression [2]. This report describes a unique case of a 52-year-old male who was diagnosed with *Mycobacterium abscessus* pulmonary disease with chronic alcohol dependence disorder as his only significant risk factor in his medical history. Medical literature has suggested alcoholism as a risk factor for NTM pulmonary infections and with this case report, we highlight the importance of understanding this association [3].

## Case presentation

A 52-year-old male with a past medical history of alcohol dependence disorder and anxiety presented to the emergency department with non-specific left upper back pain for three to four months. The pain involved the left side of the upper back and over the scapula, gradually worsening and radiating to the left axilla and left upper chest. He described the pain as sharp, intermittent, and with no aggravating factors. The patient also endorsed some unintentional weight loss, approximately 11 lbs in the past two to three months. Besides weight loss, he did not have any B-cell symptoms such as fever, night sweats, changes in appetite, or fatigue.

The patient did not have any history of incarceration or homelessness. The only traveling history for him was a cruise to the Caribbean Islands for a short period. He mostly had lived in Florida and Tennessee when he was young and was now domiciled in New York. He has a history of alcohol dependence disorder (eight to 10 beer cans daily) but no history of recreational drug use or smoking. He denies any previous history of recurrent infections, no sick contact, or contact with patients infected with tuberculosis. He did not have any history of bacillus Calmette-Guérin (BCG) vaccination or any history of opportunistic infections in the past. When he arrived in the emergency department, he was tachycardic and tachypneic but otherwise hemodynamically stable (temperature at 98.1 Fahrenheit, blood pressure 110/55 mmHg, heart rate 140, respiratory rate 32 with SpO2 74% on room air). The examination was relevant for decreased breath sound on the left lung fields and tachycardia. He was subsequently placed on high-flow nasal cannula oxygen supplementation with an improvement of his SpO2 to 94%.

**Figure 1:**
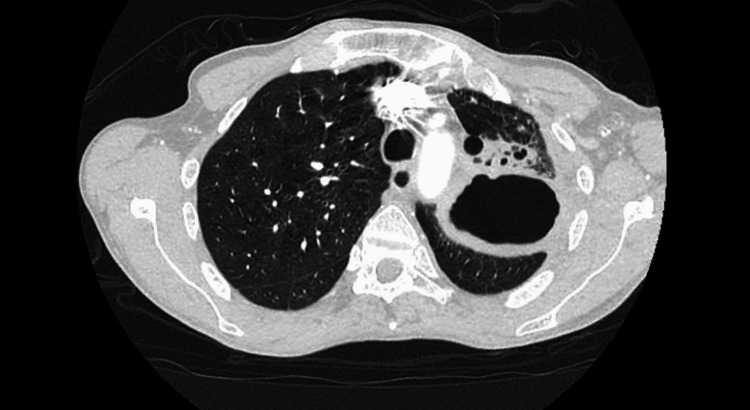
CT of the chest showing 6.8 X 5.1 X 6.7 cm cavitary lesion in the left hilum and left upper lobe

**Figure 2:**
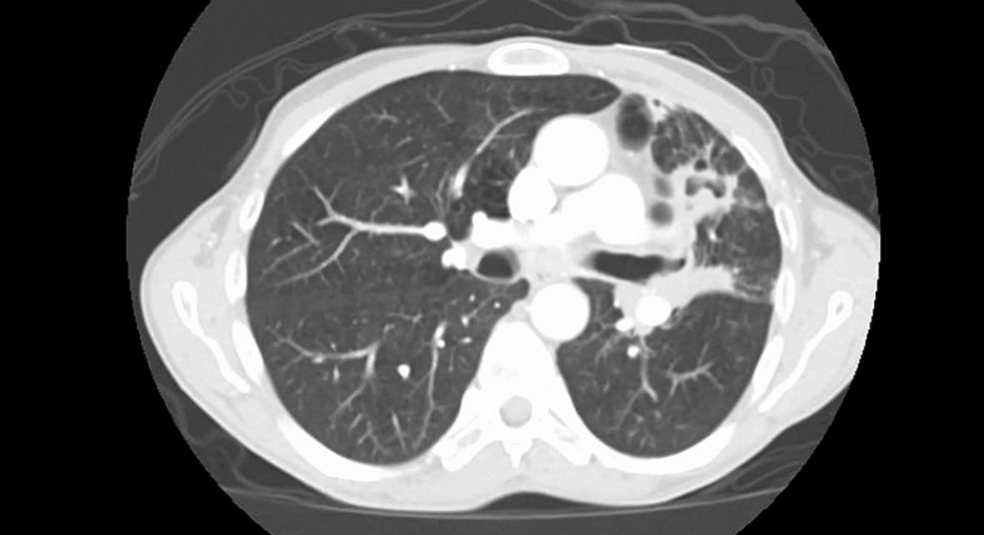
CT of the chest showing tree-in-bud opacities bilaterally

**Figure 3:**
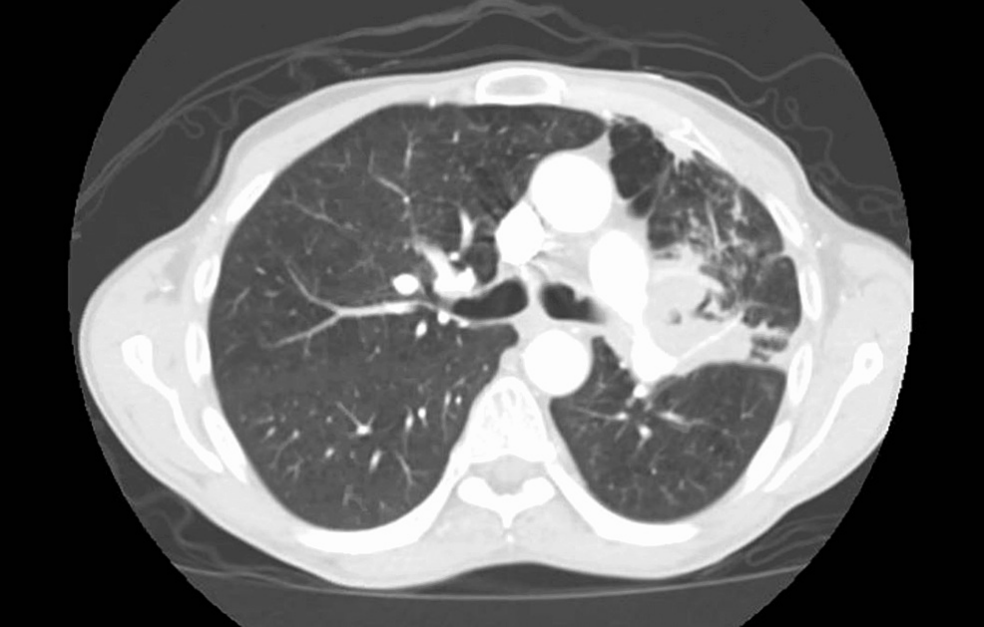
CT of the chest showing 2.7 X 1.6 X 1.3 cm cavitary lesion in the left lower lobe

He underwent emergent CT chest with intravenous contrast which revealed a 6.8 x 5.1 x 6.7 cm irregular cavitary lesion within the left hilum and left upper lobe with a 1.5 cm nodular component along the posterior aspect; severe narrowing of the left main pulmonary artery with attenuation of left upper lobe segment branch; additional smaller thick-walled cavities noted within lingula measuring up to 2.7 x 1.6 x 1.3 cm; and multiple centrilobular and tree in bud nodules within right middle lobe, lingula, and left lower lobe (Figure 1-3). He was admitted for further infectious workup.

In the workup performed, he had a purified protein derivative (PPD) done which was negative, QuantiFERON was indeterminate and HIV screen was negative. He ultimately had a bronchoscopy performed and bronchial alveolar lavage was sent for cultures which resulted in positive for *Mycobacterium abscessus* (Table 1). The patient was ultimately initiated on an intravenous antibiotic regimen with Amikacin, Tigecycline, and Cefoxitin via a peripherally inserted central catheter (PICC) line according to the susceptibility testing results, which he is planned to continue for at least six to 12 months. He will be closely followed up at the outpatient department to determine clinical improvement and resolution of the cavitary lesion with serial clinical imaging.

**Table 1:** Antibiotic susceptibilities *Mycobacterium abscessus* culture data

ANTIBIOTIC	SUSCEPTIBILITIES
Amikacin	16 μg/ml (susceptible)
Cefoxitin	64 μg/ml (intermediate)
Ciprofloxacin	>4 μg/ml (resistant)
Clarithromycin	>16 μg/ml (resistant)
Doxycycline	>16 μg/ml (resistant)
Imipenem	>32 μg/ml (resistant)
Linezolid	>32 μg/ml (resistant)
Moxifloxacin	>8 μg/ml (resistant)
Tigecycline	0.25 μg/ml (No interpretation available)
Tobramycin	16 μg/ml (resistant)
Trimethoprim-sulfamethoxazole	8 μg/ml (resistant)

## Discussion

Nontuberculous mycobacteria (NTM) have been recognized as pathogenic organisms for many years now. Although they are routinely present in the environment inhabiting the body surfaces or secretions as a colonizer, the rate of clinical infection in humans is considered to be low. As reporting is not mandatory in several countries of the world, the exact frequency of the disease due to different species of NTM is not determined. An estimated disease prevalence in the United States was approximately 15.2 cases/100,000 in 2013. It is also notable that isolation of NTM does not necessarily indicate an infection [1,4].

The infections with NTM are typically seen in immunocompromised patients, such as those with a history of prior tuberculous infection, advanced HIV disease, cystic fibrosis, and history of immunosuppressive medication intake. Diseases due to NTM are classified into broad clinical syndromes: disease of lymph nodes (lymphadenitis), skin involvement, musculoskeletal involvement, pulmonary disease, and disseminated disease based on the organ system involvement. There are over 170 identified species, however, Mycobacterium avium complex,* Mycobacterium kansasii*, *Mycobacterium abscessus*, *Mycobacterium chelonae,* and *Mycobacterium fortuitum* are most reported in infections [5,6].

Previously thought to be rare, infections with NTM are now increasingly recognized especially with the advancement of detection techniques such as polymerase chain reaction. Pulmonary involvement is considered the most common manifestation of the infection. The predisposition factors are baseline parenchymal lung diseases such as bronchiectasis, chronic obstructive pulmonary disease, interstitial lung disease, or immunosuppression in the setting of uncontrolled HIV infection, post-transplantation, and use of anti-tumor necrosis factor-α biologics. [7]

Some studies have shown that NTM pulmonary infections are noted in the post-menopausal female populations with no immune dysfunction but with slender physique, scoliosis, and mitral valve prolapse. Other less common risk factors described in the literature include gastro-esophageal reflux disease, vitamin D deficiency, low body mass index, and rheumatoid arthritis. [8]

We describe an interesting case of pulmonary *Mycobacterium abscessus* in a patient with serious alcohol dependence disorder but an otherwise immunocompetent individual. He was not noted to have any significant history to be predisposed to a lung parenchymal disease at baseline, and no history of systemic immunosuppression was noted. Theoretically, we suggest that a history of alcohol dependence disorder although not properly understood may have played some role in the predisposition to an NTM infection. Alcohol consumption has been suggested to have an altering effect on the innate and adaptive immune responses and inflammatory cascade dysregulation predisposing to viral and bacterial infections and sterile inflammation [9]. Additionally, alcohol dependence is also associated with gastroesophageal reflux disease [10]. We hypothesize that alcohol-induced reflux disease may have played some role in predisposing our patient to have a pulmonary NTM infection. The association of gastroesophageal reflux with NTM has not been studied in detail but limited data does suggest a high prevalence of such infections even in the absence of classic symptoms of reflux disease [11].

Infections by *Mycobacterium abscessus* are classically considered to be difficult to manage with no standard treatment. The treatment options are limited with current antimicrobial agents and hence is often considered a chronic incurable infection in the right clinical setting. [12]

## Conclusions

Non-tuberculous mycobacterium is an important environmental pathogen that may cause a broad spectrum of diseases in humans. It is important to elucidate important risk factors including immune system dysregulation which may predispose to significant infections by these organisms. Alcoholism is considered to be an important risk factor for predisposing patients to infections with NTM and with this case report, we emphasize doing more robust studies to evaluate this association.
